# The Comparison of Surgical Margins and Type of Hepatic Resection for Hepatocellular Carcinoma With Microvascular Invasion

**DOI:** 10.1093/oncolo/oyad124

**Published:** 2023-05-17

**Authors:** Jianwei Liu, Guokun Zhuang, Shilei Bai, Zhiliang Hu, Yong Xia, Caixia Lu, Jie Wang, Chunyan Wang, Liu Liu, Fengwei Li, Yeye Wu, Feng Shen, Kui Wang

**Affiliations:** Department of Hepatic Surgery II, Third Affiliated Hospital of Naval Medical University (Eastern Hepatobiliary Surgery Hospital), Shanghai, People’s Republic of China; Department of Hepatic Surgery II, Third Affiliated Hospital of Naval Medical University (Eastern Hepatobiliary Surgery Hospital), Shanghai, People’s Republic of China; Department of Hepatic Surgery II, Third Affiliated Hospital of Naval Medical University (Eastern Hepatobiliary Surgery Hospital), Shanghai, People’s Republic of China; Department of Hepatic Surgery II, Third Affiliated Hospital of Naval Medical University (Eastern Hepatobiliary Surgery Hospital), Shanghai, People’s Republic of China; Department of Hepatic Surgery IV, Third Affiliated Hospital of Naval Medical University (Eastern Hepatobiliary Surgery Hospital), Shanghai, People’s Republic of China; Department of Hepatic Surgery II, Third Affiliated Hospital of Naval Medical University (Eastern Hepatobiliary Surgery Hospital), Shanghai, People’s Republic of China; Department of Hepatic Surgery II, Third Affiliated Hospital of Naval Medical University (Eastern Hepatobiliary Surgery Hospital), Shanghai, People’s Republic of China; Department of Hepatic Surgery II, Third Affiliated Hospital of Naval Medical University (Eastern Hepatobiliary Surgery Hospital), Shanghai, People’s Republic of China; Department of Hepatic Surgery II, Third Affiliated Hospital of Naval Medical University (Eastern Hepatobiliary Surgery Hospital), Shanghai, People’s Republic of China; Department of Hepatic Surgery II, Third Affiliated Hospital of Naval Medical University (Eastern Hepatobiliary Surgery Hospital), Shanghai, People’s Republic of China; Department of Hepatic Surgery II, Third Affiliated Hospital of Naval Medical University (Eastern Hepatobiliary Surgery Hospital), Shanghai, People’s Republic of China; Department of Hepatic Surgery IV, Third Affiliated Hospital of Naval Medical University (Eastern Hepatobiliary Surgery Hospital), Shanghai, People’s Republic of China; Department of Hepatic Surgery II, Third Affiliated Hospital of Naval Medical University (Eastern Hepatobiliary Surgery Hospital), Shanghai, People’s Republic of China; Department of Hepatic Surgery II, Third Affiliated Hospital of Naval Medical University (Eastern Hepatobiliary Surgery Hospital), Shanghai, People’s Republic of China

**Keywords:** hepatocellular carcinoma, anatomical resection, nonanatomical resection, surgical margin, microvascular invasion, prognosis

## Abstract

**Objective:**

The objective of this study was to investigate the impact of surgical margin and hepatic resection on prognosis and compare their importance on prognosis in patients with hepatocellular carcinoma (HCC).

**Methods:**

The clinical data of 906 patients with HCC who underwent hepatic resection in our hospital from January 2013 to January 2015 were collected retrospectively. All patients were divided into anatomical resection (AR) (*n* = 234) and nonanatomical resection (NAR) group (*n* = 672) according to type of hepatic resection. The effects of AR and NAR and wide and narrow margins on overall survival (OS) and time to recurrence (TTR) were analyzed.

**Results:**

In all patients, narrow margin (1.560, 1.278-1.904; 1.387, 1.174-1.639) is an independent risk factor for OS and TTR, and NAR is not. Subgroup analysis showed that narrow margins (2.307, 1.699-3.132; 1.884, 1.439-2.468), and NAR (1.481, 1.047-2.095; 1.372, 1.012-1.860) are independent risk factors for OS and TTR in patients with microvascular invasion (MVI)-positive. Further analysis showed that for patients with MVI-positive HCC, NAR with wide margins was a protective factor for OS and TTR compared to AR with narrow margins (0.618, 0.396-0.965; 0.662, 0.448-0.978). The 1, 3, and 5 years OS and TTR rate of the two group were 81%, 49%, 29% versus 89%, 64%, 49% (*P* = .008) and 42%, 79%, 89% versus 32%, 58%, 74% (*P* = .024), respectively.

**Conclusions:**

For patients with MVI-positive HCC, AR and wide margins were protective factors for prognosis. However, wide margins are more important than AR on prognosis. In the clinical setting, if the wide margins and AR cannot be ensured at the same time, the wide margins should be ensured first.

## Teaser

This study investigated the impact of surgical margin and hepatic resection on prognosis for patients with hepatocellular carcinoma.

**Implications for Practice**

For patients with microvascular invasion (MVI)-positive hepatocellular carcinoma (HCC), anatomical resection and wide margins were protective factors for overall survival and time to recurrence; however, wide margins are more important than anatomical resection on prognosis for patients with MVI-positive HCC. Anatomical resection should be performed under the condition of ensuring wide margins. In the clinical setting, if the wide margins and anatomical resection cannot be ensured at the same time, the wide margins should be ensured first.

## Introduction

Hepatocellular carcinoma (HCC) is the most common type of primary liver cancer, and its incidence is increasing every year. In China, it has become the third leading cause of ­cancer-related death.^1^ Hepatectomy is the most important and effective treatment for patients with HCC, but the high recurrence rate after hepatectomy lead to unsatisfactory ­prognosis.^1-4^ A growing number of studies suggested that microvascular invasion (MVI) is an important indicator of tumor aggressiveness and malignancy and plays an important role in postoperative recurrence in patients with HCC.^3,5-7^ MVI is defined as a cluster of cancer cells found in the microscopic endothelial cell-lined vascular lumen, which occurs mainly in the portal venous system.^8^ Different patients with HCC have different etiologies and pathogenic mechanisms that lead to different proportions of MVI in different patients with HCC. Studies had reported MVI-positive rates ranging from 11% to 60% in patients with HCC.^9^ How to reduce the high rate of tumor recurrence and improve the overall survival (OS) for MVI-positive patients with HCC are currently a hotspot issue in clinical research.

Previous studies showed that anatomical resection (AR) can reduce the tumor recurrence rate and improve the prognosis in patients with MVI-positive.^10-12^ Wide margins can also reduce postoperative tumor recurrence rates and improve prognosis compared to narrow margins in patients with MVI-positive.^13^ However, there is still controversy regarding the prognostic impact of hepatic resection and surgical margins in patients with MVI-positive. There is also a lack of studies comparing the hepatic resection and surgical margins in patients with MVI-positive. In this study, 906 patients with HCC were included. The effect of hepatic resection and surgical margins on the prognosis of patients with MVI-positive HCC was investigated.

## Patients and Methods

### Patients

Patients with HCC who underwent hepatectomy in our hospital from January 2013 to January 2015 were collected retrospectively. All patients included in this study underwent open surgery. The patients were selected according to the inclusion and exclusion criteria, and detailed clinical information of the patients was recorded. The inclusion criteria for this study were: (a) HCC was confirmed by postoperative pathological, (b) no extrahepatic distant metastasis, (c) no macrovascular invasion and no invasion of peripheral organs, (d) complete resection of tumor with negative surgical margins, and (e) no other anti-tumor treatments prior to hepatectomy.

Exclusion criteria were: (a) severe cardiopulmonary dysfunction, unable to tolerate hepatectomy; (b) Child-Pugh score beyond B7, clinically significant portal hypertension^14^; (c) undergoing non-R0 resection^15^; (d) postoperative pathologically confirmed intrahepatic cholangiocarcinoma (ICC) or mixed HCC-ICC; (e) intraoperative procedures other than hepatectomy that may affect postoperative complications, eg, biliary-intestinal anastomosis; (f) incomplete clinical data; and (g) lost in follow-up within one month after surgery.

### Preoperative Examination and Hepatectomy

Preoperative examination is routinely performed to assess the patient’s surgical tolerance and resectability of the tumors. Preoperative examination included: blood routine, hepatic and renal function, coagulation function, tumor markers, hepatitis markers, blood grouping, electrocardiogram (ECG), lung function, gastroscopy, chest Computed Tomography (CT), abdominal ultrasound, and liver Magnetic Resonance Imaging (MRI).

All patients included in this study underwent AR or NAR and major hepatectomy or minor hepatectomy according to different hepatectomy methods. AR^16^: AR was charactered as any type of complete excision at least one segment based on Couinaud’s classification, included segmentectomy, subsegmentectomy, sectoriectomy, and hemihepatectomy. After laparotomy, the liver was completely exposed, and the perihepatic ligament was released. According to the preoperative imaging combined with intraoperative ultrasound, the anatomy of the liver and the corresponding segment or lobe which should be resected was confirmed. The intraoperative ultrasound is used to identify the intrahepatic veins, hepatic artery, and bile ducts, and the corresponding liver segment is defined by combination with the hepatic vein and portal vein. The liver parenchyma is then dissected, and the intrahepatic veins, hepatic artery or bile ducts are dissected by electrocoagulation, ligation, and suturing, respectively. Then, the corresponding liver segment was completely resected. After complete resection of the tumors, the liver section is completely hemostasis. NAR^17^: The same exposure process as AR, then according to the position of the tumor, the resection line is set by electric knife in advance on the surface of the liver. The tumor was completely resected by electrocoagulation, ligation, and suturing, respectively, along the resection line. After complete resection of the tumors, the liver section is completely hemostasis. Major hepatectomy and minor hepatectomy were classified according to the extent of hepatectomy. Hepatectomy ≥3 liver segments is defined as major hepatectomy,^18,19^ and hepatectomy <3 liver segments is defined as minor hepatectomy.

Based on the postoperative pathologic, wide or narrow margin was defined as the shortest distance from the margin of tumors to the surgical margin ≥ 1 cm or not.^13,20^

### Follow-Up and Endpoints

Tumor differentiation was graded by postoperative pathology according to the Edmondson-Steiner classiﬁcation.^21^ Postoperative complications were assessed according to the Clavien-Dindo criteria.^22^ In this study, some patients returned to the hospital for adjuvant TACE about one month after surgery. The screening of these patients is based on factors such as tumor size, number of tumors, and MVI. If the patient is assessed as a high-risk recurrence after surgery, it is generally recommended that patients undergo an adjuvant TACE about one month after surgery.^23^ Routine postoperative follow-up was performed every 2-3 months for first 2 years and every 3-6 months after 2 years. Blood routine, hepatic and renal function, tumor markers, abdominal ultrasound, liver MRI, or CT was performed at follow-up. The American Association for the Study of Liver Diseases (AASLD) criteria were used for the diagnosis of HCC recurrences.^24^ OS and TTR were used as the primary endpoints. OS was defined as the day of hepatic resection until the patients died or lost to follow-up. TTR was defined as the day of hepatic resection until tumor recurrences or metastasis.

### Statistical Analysis

The measure data were described by median (range), and independent samples *t* test or Mann-whitney *U* test were used to evaluate the statistical differences. The Kaplan-Meier method was used to plot survival and recurrence curves. And the Cox’s univariate and multivariate analysis was used to evaluate the independent risk factors for OS and TTR. Age, sex, alpha fetoprotein (AFP) levels, tumor diameter, tumor number, MVI, tumor capsule, and Edmondson-Steiner grade were selected for the subgroup analysis. Hazard Ratio (HR) and 95% confidence interval (CI) represent relative risk, with *P*<.05 considered a statistically significant difference. All data analysis was performed by SPSS software 26.0 (Statistical Program for Social Sciences Inc., Chicago, IL, USA).

## Results

### Patient Characteristics

**Figure 1.**
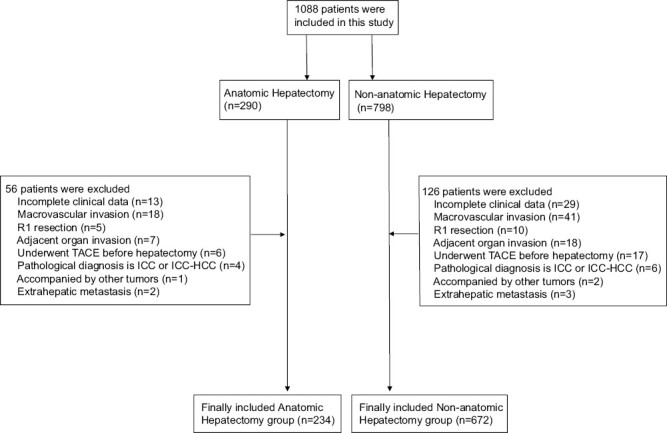
The flow chart of this study.

**Fig. 1** shows the flow chart of this study, with 906 patients with HCC eventually included in this study. **Supplementary Table S1** shows basic information about the 906 patients. According to patients underwent AR or NAR, 906 patients were divided into AR group (*n* = 234) and NAR group (*n =* 672). There was no statistical difference between the two groups in sex, age, Body Mass Index (BMI), diabetes, hepatitis B surface antigen (HBsAg), hepatitis Be antigen (HBeAg), hepatitis C virus (HCV), hepatitis B virus (HBV)-DNA, preoperative antiviral therapy, total bilirubin (TBIL), alanine aminotransferase (ALT), platelet (PLT), AFP levels, blood transfusion, cirrhosis, tumor diameter, tumor number, tumor capsule, MVI, Edmondson-Steiner grade, postoperative complications, and adjuvant TACE (*P* > .05). The AR group compared with NAR group had higher albumin (ALB) levels (41.1 g/L vs. 40.1 g/L, *P* = .007), lower prothrombin time (PT) levels (12.0 seconds vs. 12.2 seconds, *P* = .023), more wide margins (56.0% vs. 47.5%, *P* = .025), major hepatectomy (35.9% vs. 27.7%, *P* = .018), and hilar clamping > 20 minutes (77.8% vs. 70.5%, *P* = .033).

### OS and TTR in the Whole Cohort

The median follow-up time of the 906 patients with HCC was 63.7 months, with the 1, 3, and 5 years OS rates, and tumor recurrence rates were 87.2%, 63.5%, 49.2% and 21.7%, 55.6%, 68.6%, respectively.

**Figure 2.**
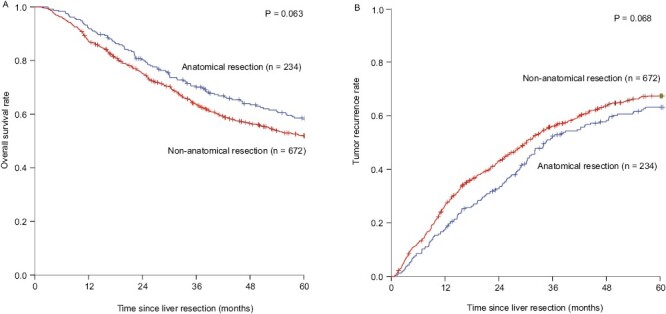
Kaplan-Meier estimate of OS and tumor recurrence for anatomical resection and nonanatomical resection group in the whole group. (**A**) Kaplan-Meier estimate of OS for patients with HCC underwent anatomical resection and nonanatomical resection; (**B**) Kaplan-Meier estimate of tumor recurrence for patients with HCC underwent anatomical resection and nonanatomical resection.

The results of the univariate analysis are shown in **Supplementary Table S2**. All variables had statistically difference in univariate analysis were included in the multivariate analysis. The results showed that AFP > 200 ng/mL (1.465, 1.168-1.838; 1.224, 1.017-1.473), tumor diameter >5 cm (2.048, 1.660-2.526; 1.640, 1.382-1.947), multiple tumors (1.901, 1.549-2.334; 1.820, 1.518-2.182), tumor capsule incomplete (1.576, 1.269-1.955; 1.386, 1.157-1.659), MVI (1.718, 1.398-2.113; 1.461, 1.224-1.745) were independent risk factors for OS and TTR (**Table 1**). AR was not an independent risk factor for OS and TTR, and the 1, 3, and 5 years OS rate was 91.0%, 67.5%, 50.5% and 85.8%, 62.0%, 48.7% in AR and NAR group (*P* = .063), respectively (**Fig. 2A**). The 1, 3, and 5 years tumor recurrence rates were 13.7%, 53.2%, 67.0% and 24.5%, 56.4% and 69.2% in AR and NAR group (*P* = .068), respectively (**Fig. 2B**).

**Table 1.** Multivariate analysis of OS and TTR.

Variable	OS			TTR		
	*P*	HR	95% CI	*P*	HR	95% CI
HBV-DNA, IU/mL, > vs. ≤ 2000	——	—	—	.052	1.184	0.999—1.403
PLT, 10^9^/L, ≤ vs. > 100	.257	0.853	0.649—1.122	.118	0.837	0.669—1.046
AFP, ng/mL, > vs. ≤ 200	.001	1.465	1.168—1.838	.033	1.224	1.017—1.473
Blood transfusion, yes vs. no	.339	1.160	0.855—1.574	—	—	—
Tumor diameter^§^, cm, > vs. ≤ 5	<.001	2.048	1.660-2.526	<.001	1.640	1.382-1.947
Tumor number^§^, multiple^†^ vs. single	<.001	1.901	1.549-2.334	<.001	1.820	1.518-2.182
Surgical margin^§^, cm, ≤ vs. >1.0	<.001	1.560	1.278-1.904	<.001	1.387	1.174-1.639
Tumor capsule^§^, incomplete vs. complete	<.001	1.576	1.269-1.955	<.001	1.386	1.157-1.659
MVI^§^, presence vs. absence	<.001	1.718	1.398-2.113	<.001	1.461	1.224-1.745
Edmondson-Steiner grade^§^, III/IV vs. I/II	.182	1.201	0.918-1.573	.199	1.151	0.929-1.426

^§^Based on postoperative pathology.

^†^Tumor nodules ≥ 2.

Abbreviations: OS: overall survival; HR: hazard ratio; CI: Confidence interval; TTR: time to recurrence; HBV-DNA: hepatitis B virus deoxyribonucleic acid; PLT: platelet; AFP: alpha fetoprotein; MVI: microvascular invasion.

### OS and TTR in the Patients With MVI-Negative HCC

The 906 patients with HCC were divided into MVI-negative (*n* = 588) and MVI-positive (*n* = 318) groups.

**Supplementary Table S3** shows the differences of basic information between AR group and NAR group in patients with MVI-negative HCC. **Supplementary Table S4** shows the results of univariate analysis for OS and TTR. Multivariate analysis shows that AFP >200 ng/mL (1.468, 1.081-1.994; 1.341, 1.067-1.685), tumor diameter >5 cm (2.161, 1.626-2.872; 1.703, 1.368-2.120), multiple tumors (2.032, 1.517-2.723; 2.067, 1.618-2.640), and tumor capsule incomplete (1.648, 1.248-2.176; 1.502, 1.205-1.872) were independent risk factors for OS and TTR (**Table 2**). The hepatic resection and surgical margins were not influencing factors for OS and TTR in patients with MVI-negative HCC.

**Table 2.** Multivariate analysis of OS and TTR of patients with MVI-negative and MVI-positive HCC.

Variable	OS			TTR		
	*P*	HR	95% CI	*P*	HR	95% CI
MVI-negative						
Blood transfusion, yes vs. no	.202	1.323	0.861-2.032	—	—	—
AFP, ng/mL, > vs. ≤ 200	.014	1.468	1.081-1.994	.012	1.341	1.067-1.685
Tumor diameter^§^, cm, > vs. ≤ 5	<.001	2.161	1.626-2.872	<.001	1.703	1.368-2.120
Tumor number^§^, multiple^†^ vs. single	<.001	2.032	1.517-2.723	<.001	2.067	1.618-2.640
Tumor capsule^§^, incomplete vs. complete	<.001	1.648	1.248-2.176	<.001	1.502	1.205-1.872
Edmondson-Steiner grade^§^, III/IV vs. I/II	.522	1.117	0.797—1.566	-	-	-
MVI-positive						
PLT, 10^9^/L, ≤ vs. > 100	.671	.919	0.621—1.359	.190	.793	0.561-1.121
HBV-DNA, IU/mL, > vs. ≤ 2000	—	—	—	.004	1.466	1.133-1.897
AFP, ng/mL, > vs. ≤ 200	.037	1.429	1.021-2.001	.456	1.116	0.836-1.489
Tumor diameter^§^, cm, > vs. ≤ 5	<.001	1.971	1.450-2.680	<.001	1.660	1.266-2.176
Tumor number^§^, multiple^†^ vs. Single	.001	1.623	1.214-2.169	.007	1.452	1.110-1.900
Surgical margin^§^, cm, ≤ vs. >1.0	<.001	2.307	1.699-3.132	<.001	1.884	1.439-2.468
Hepatectomy, anatomic vs. nonanatomic	.026	1.481	1.047-2.095	.042	1.372	1.012-1.860
Edmondson-Steiner grade^§^, III/IV vs. I/II	.412	1.210	0.767-1.911	.274	1.246	0.840-1.847

^§^Based on postoperative pathology.

^†^Tumor nodules ≥ 2.

Abbreviations: OS: overall survival; HR: hazard ratio; CI: Confidence Interval; TTR: time to recurrence; AFP: alpha fetoprotein; MVI: microvascular invasion; PLT: platelet; HBV-DNA: hepatitis B virus deoxyribonucleic acid.

**Figure 3.**
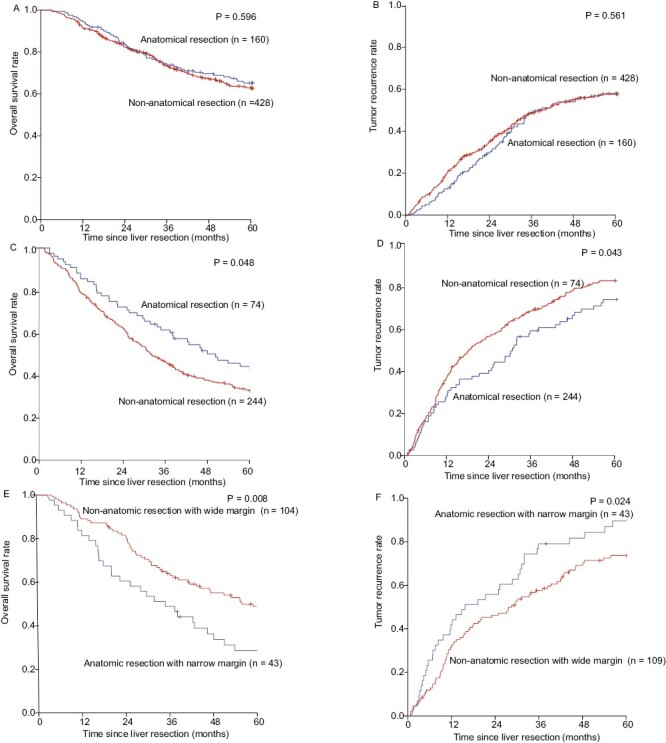
Kaplan-Meier estimate of OS and tumor recurrence for patients with MVI-negative and MVI-positive HCC underwent anatomical resection and nonanatomical resection and for patients with MVI-positive HCC underwent anatomical resection with narrow margin and nonanatomical resection with wide margin. (**A**) Kaplan-Meier estimate of OS for patients with MVI-negative HCC underwent anatomical resection and non-anatomical resection; (**B**) Kaplan-Meier estimate of tumor recurrence for patients with MVI-negative HCC underwent anatomical resection and nonanatomical resection; (**C**) Kaplan-Meier estimate of OS for patients with MVI-positive HCC underwent anatomical resection and nonanatomical resection; (**D**) Kaplan-Meier estimate of tumor recurrence for patients with MVI-positive HCC underwent anatomical resection and nonanatomical resection. (**E**) Kaplan-Meier estimate of OS for patients with MVI-positive HCC underwent anatomical resection with narrow margin and nonanatomical resection with wide margin; (**F**) Kaplan-Meier estimate of tumor recurrence for patients with MVI-positive HCC underwent anatomical resection with narrow margin and nonanatomical resection with wide margin.

The 1, 3, and 5 years OS rate of AR group and NAR group was 94.4%, 73.7%, 65.2%, and 91.6%, 73.6%, 62.9% (*P* = .596), respectively. And the 1, 3, and 5 years tumor recurrence rate were 12.5%, 49.0%, 57.7% and 20.2%, 48.7%, 58.0% (*P* = .561), respectively (**Fig. 3A and 3B**).

### OS and TTR in the Patients With MVI-Positive HCC

**Supplementary Table S5** shows that there was no difference in all basic information between AR group and NAR group in patients with MVI-positive HCC (*P* > 0.05). **Supplementary Table S6** shows the results of univariate analysis for OS and TTR in patients with MVI-positive HCC. The results of the multivariate analysis are shown in **Table 2**, which showed that tumor diameter >5 cm (1.971, 1.450-2.680; 1.660, 1.266-2.176), multiple tumors (1.623, 1.214-2.169; 1.452, 1.110-1.900), narrow margins (2.307 1.699-3.132; 1.884, 1.439-2.468), NAR (1.481, 1.047-2.095; 1.372, 1.012-1.860) were independent risk factors for OS and TTR. And AFP >200 ng/mL (1.429, 1.021-2.001) was an independent risk for OS. HBV-DNA >2000 IU/mL (1.466, 1.133-1.897) was an independent risk factor for TTR.

The 1, 3, and 5 years OS rate of AR and NAR group was 86.5%, 62.1%, 44.6% and 79.5%, 47.0%, 33.5% (*P* = .048), respectively. And the 1, 3, and 5 years tumor recurrence rates of AR and NAR group were 28.4%, 59.7%, 74.5% and 37.2%, 68.9%, 83.3%, (*P* = .043) (**Fig. 3C and 3D**).

### Comparison of the Importance of Hepatic Resection and Surgical Margin for Patients with MVI-Positive HCC

**Supplementary Table S7** shows that there was no difference in all basic information between AR combined with narrow margins and NAR combined with wide margins in patients with MVI-positive HCC (*P* > .05). The results of the univariate analysis are shown in **Supplementary Table S8**.

The results of the multivariate analysis showed that tumor diameter >5 cm (1.610, 1.013-2.561) and multiple tumors (1.684, 1.072-2.646) were independent risk factors for OS. Tumor diameter >5 cm (1.541, 1.057-2.245) was an independent risk factor for TTR. NAR combined with wide margins (0.618, 0.396-0.965; 0.662, 0.448-0.978) was a protective factor for OS and TTR compared with AR combined with narrow margins (**Table 3**).

**Table 3.** Multivariate analysis of OS and TTR of patients with MVI-positive HCC underwent AR with narrow margin or NAR with wide margin.

Variable	OS			TTR		
	*P*	HR	95% CI	*P*	HR	95% CI
AFP, ng/mL, > vs. ≤ 200	.201	1.397	0.837-2.333	—	—	—
Hepatectomy, AR with narrow margin vs. NAR with wide margin	.034	.618	0.396-0.965	.038	.662	0.448—0.978
Tumor diameter^§^, cm, > vs. ≤ 5	.044	1.610	1.013-2.561	.024	1.541	1.057—2.245
Tumor number^§^, multiple^†^ vs. Single	.024	1.684	1.072-2.646	—	—	—

^§^Based on postoperative pathology.

^†^Tumor nodules ≥ 2.

Abbreviations: AR: anatomical resection; NAR: nonanatomical resection; OS: overall survival; HR: hazard ratio; CI: Confidence interval; TTR: time to recurrence; AFP: alpha fetoprotein; MVI: microvascular invasion.

The 1, 3, and 5 years OS rate was 81.4%, 48.8%, 28.8% and 89.0%, 63.8%, 48.7% for AR combined with narrow margins and NAR combined with wide margins (*P* = .008), respectively. The 1, 3, and 5 years TTR rates was 41.9%, 79.1%, 89.5% and 32.3%, 57.8%, 73.7% (*P* = .024), respectively (**Fig. 3E and 3F**).

### Complications and Mortality

The incidence of all grade postoperative complications rates were 178/906 (19.6%) and 66/906 (7.2%) in grade III/IV postoperative complications. The all grade postoperative complications were 51/234 (21.8%) in AR group and 127/672 (18.9%) in NAR group (*P* = .337). The grade III/IV complications rates were 19/234 (8.1%) in AR group and 47/672 (7.0%) in NAR group (*P* = .568). There was no significant difference in all grade postoperative complications and grade III/IV complications between the two groups (**Table 4**).

**Table 4.** Complications of patients underwent AR and NAR.

Complications	All grade (*n*)^*^			Grade III/IV (*n*) ^*^		
	AR group (*n* = 234)	NAR group (*n* = 672)	*P*	AR group (*n* = 234)	NAR group (*n* = 672)	*P*
Number of patients	51(21.8)^**^	127(18.9)^#^	.337	19(8.1)^***^	47(7.0)^##^	.568
Overall complication events	87 (100%)	228(100%)		25(100%)	66(100%)	
Hepatic insufficiency^###^	10 (11.5%)	24 (10.5%)		4 (16.0%)	9 (13.6%)	
Pleural effusion	8 (9.2%)	25 (11.0%)		2 (8.0%)	10 (15.2%)	
Ascites	12 (13.8%)	31 (13.6%)		4 (16.0%)	12 (18.2%)	
Fever (> 38.5°C, > 3 days)	23(26.4%)	58 (25.4%)		5 (20.0%)	14 (21.2%)	
Intra-abdominal hemorrhage	6 (6.9%)	14 (6.1%)		3 (12.0%)	6 (9.1%)	
Intra-abdominal infection	3 (3.5%)	8 (3.5%)		2 (8.0%)	3 (4.5%)	
Bile leakage	8 (9.2%)	19 (8.3%)		2 (8.0%)	4 (6.1%)	
Pneumonia	5 (5.7%)	12 (5.3%)		1 (4.0%)	3 (4.5%)	
Wound infection	6 (6.9%)	22 (9.6%)		0	0	
Gastrointestinal hemorrhage	3 (3.5%)	10 (4.4%)		1 (4.0%)	3 (4.5%)	
Others	3 (3.4%)	5 (2.3%)		1 (4.0%)	2 (3.1%)	

^*^According to the Clavien-Dindo classification.

^**^20 patients occurred 1 complication, 26 patients occurred 2 different complications, 5 patient occurred 3 different complications.

^***^13 patients occurred 1 complication, 6 patients occurred 2 different complications, 1 patient occurred, 3 different complications.

^#^44 patients occurred 1 complication, 65 patients occurred 2 different complications, 18 patient occurred 3 different complications.

^##^30 patients occurred 1 complication, 15 patients occurred 2 different complications, 2 patient occurred 3 different complications.

^###^Liver dysfunction was defined using the “50–50” criteria.

Abbreviations: AR, anatomical resection; NAR, non-anatomical resection.

## Discussion

Hepatectomy is the most important and effective treatment for patients with HCC to obtain radical treatment.^4^ Depending on the hepatic resection, hepatectomy can be divided into AR and NAR.^25,26^ AR is the complete resection of the tumors and associated portal branches, and the corresponding at least one liver segment.^27^ AR can not only removes tumors that are visible to naked eye but also removes MVI that is difficult to detect before hepatectomy.^28^ In addition, AR can completely remove the tumor-carrying portal tributaries and reduce the ischemic area after surgery.^29^ With the development of the concept of precision surgery, AR is receiving more and more attention.^10-12^ However, the impact of AR on prognosis remains controversial.^10-12,26,30,31^ Some studies suggested that AR does not improve the prognosis of patients with HCC.^26,30,31^ In contrast, those who supported AR suggest that AR can remove the liver segment and the corresponding portal vein basin together with the intrahepatic lesion, which theoretically minimizes the risk of tumor dissemination and metastasis in the liver segment by the tumor-bearing basin with portal blood flow, thereby reducing postoperative tumor recurrence and improving surgical outcomes.^10-12^

The results of our research showed that AR did not affect the prognosis of patients with HCC in the whole group, which is consistent with the results of previous studies.^13,31^ However, in patients with MVI-positive, AR improved patient prognosis and reduced tumor recurrence compared to NAR. Previous studies have reported that AR in MVI-positive patients reduces postoperative tumor recurrence,^10^ and improves patients’ recurrence free survival (RFS)^12,32^ and OS.^11,33-36^ This may be related to the fact that AR can remove intrahepatic lesions and microvascular metastases, which can reduce postoperative tumor recurrence.^12,33^ In addition, AR can reduce the rate of early tumor recurrence and intrahepatic recurrence in the adjacent surgical area, which is also related to the fact that AR can more effectively remove the intrahepatic micrometastases.^12^ Furthermore, the results of our research showed that AR had higher proportion of wide margins compared to NAR, which is consistent with previous studies reported.^12^ Previous studies have reported that wide margins can significantly improve patient survival compared to narrow margins.^13,37^ Although previous studies have not explicitly proposed that a better prognosis of AR may be related to a higher proportion of wide margins. Based on our results, we believed that a better prognosis of AR may be related to a higher proportion of wide margins. Our study also analyzed the impact of surgical margins on prognosis. The results showed that wide margins can improve the prognosis of patients in the whole group, which is consistent with previous studies reported.^13,37^ Further subgroup analysis showed that in patients with MVI-positive, wide margin can improve the prognosis which is consistent with previous studies reported.^13^ This may due to the fact that narrow margins could lead to residual MVI or residual micrometastases, which can cause intrahepatic metastases or early tumor recurrence.^13^ It was found that, although MVI is mainly found in intra-microvessel, it can also invade beyond the capsules of HCC.^38^ In contrast, wide margins can remove residual MVI-induced intrahepatic micrometastases, thereby improving the prognosis.

The results of our study also showed that wide margins and AR should be advocated in patients with MVI-positive. However, some patients with HCC have insufficient residual liver volume to obtain AR and wide margins at the same time. In this case, should we give priority to AR or wide margins? Our study showed that for patients with MVI-positive, AR with narrow margins was an independent risk factor for OS and TTR compared with NAR with wide margins. In other words, although both wide margins and AR can improve the prognosis of patients with MVI-positive HCC, the wide margins had a greater impact on patients’ prognosis compared with AR. Besides, we also did some additional data analysis in our study. For patients with MVI-positive, the 1-, 3-, 5-year OS rate and TTR rate of AR with wide margins and AR with narrow margins were 93.5%, 80.5%, 66.6% versus 81.4%, 48.8%, 28.8% (*P* < .001) and 9.7%, 32.6%, 53.6% versus 41.9%, 79.1%, 89.5% (*P* < .001), respectively. There were significant differences in OS and TTR between the two groups. For patients with MVI-positive, the 1-, 3-, 5-year OS rate and TTR rate of NAR with wide margins and NAR with narrow margins were 89.0%, 63.8%, 48.7% versus 71.9%, 33.3%, 21.1% (*P* < .001) and 32.3%, 57.8%, 73.3% versus 42.0%, 78.1%, 91.0% (*P* < .001), respectively. There were significant differences in OS and TTR between the two groups. For patients with MVI-positive, the 1-, 3-, 5-year OS rate and TTR rate of AR with wide margins and NAR with wide margins were 93.5%, 80.5%, 66.6% versus 89.0%, 63.8%, 48.7% (*P* = .062) and 9.7%, 32.6%, 53.6% versus 32.3%, 57.8%, 73.7% (*P* = .012), respectively. There was significant differences in TTR between the two groups. But there is no difference in OS. From the analysis of the above subgroups, we can also find that among patients with MVI-positive, patients with wide margins receiving AR or NAR have better OS and TTR than patients with narrow margins, with significant differences. However, for patients with MVI-positive and wide margins, there is no statistical difference in OS between AR and NAR. This also suggests that for patients with MVI-positive, wide margins may be more important than AR for the prognosis of patients. Therefore, when wide margins and AR cannot be obtained at the same time in patients with MVI-positive, wide margins should be ensured firstly to obtain a better long-term prognosis.

We also analyzed the differences in cirrhosis between the AR group and NAR group in our study. Our results showed that the proportion of cirrhosis in patients with NAR is relatively high, but there is no significant difference in cirrhosis between the AR group and NAR group (43.2% vs. 46.7%, *P* = .346). This may be due to the inclusion of patients in this study who underwent rigorous liver function assessment and screening before surgery. The liver function of the patients included in this study is beyond B7 of Child-Pugh. The overall condition of liver function in these patients included in this study is good. In addition, all patients underwent evaluated for residual liver volume before surgery. It may be due to these reasons that clinicians have relatively few concerns about liver injury when deciding on surgical procedures. For patients with HCC with cirrhosis or fibrosis, clinicians will also perform AR.

Of course, this study was a single center retrospective study. Although the number of patients included in this study reached 906. The number of patients in some subgroups is relatively small during subgroup analysis. Besides, all patients included in this study come from one hospital. There may be some selection bias. Therefore, in the next step, we plan to conduct this research through multicenter.

## Conclusion

Clinically, surgical margins are more important for the prognosis of patients with HCC than the type of hepatic resection. For patients with MVI-positive HCC, both AR and wide margins are protective factors for prognosis. However, wide margins are more important for the prognosis than AR. Therefore, AR should be pursued only if wide margins are secured. In the clinical setting, if only one of the wide margins and AR can be selected, the wide margins should be ensured first.

## Supplementary Material

oyad124_suppl_Supplementary_Table_1

oyad124_suppl_Supplementary_Table_2

oyad124_suppl_Supplementary_Table_3

oyad124_suppl_Supplementary_Table_4

oyad124_suppl_Supplementary_Table_5

oyad124_suppl_Supplementary_Table_6

oyad124_suppl_Supplementary_Table_7

oyad124_suppl_Supplementary_Table_8

## Data Availability

The data supporting the findings of this study are available upon request from the corresponding author. The data are not publicly available due to privacy or ethical restrictions.
