# Supplementary material for: The Comparison of Surgical Margins and Type of Hepatic Resection for Hepatocellular Carcinoma With Microvascular Invasion
Source: Oncologist. 2023 May 17;28(11):e1043–51. doi: 10.1093/oncolo/oyad124 (PMC10628578; doi:10.1093/oncolo/oyad124)
Supplement: oyad124_suppl_Supplementary_Table_1 [file oyad124_suppl_supplementary_table_1.docx]

**Supplement Table 1. Baseline characteristics of patients**

| **Variable** | **Number (%)/median (range)** | | ***P*** |
| --- | --- | --- | --- |
|  | **AR group**  **(n=234)** | **NAR group**  **(n=672)** |  |
| Sex, male | 195 (83.3) | 551 (82.0) | 0.644 |
| ***Initial hepatectomy stage data*** |  |  |  |
| Age, years | 53.5 (18.0-79.0) | 52.0 (18.0-80.0) | 0.351 |
| BMI, ≥ 24 kg/m^2^ | 66 (28.2) | 163 (24.3) | 0.231 |
| Diabetes, yes | 10 (4.3) | 50 (7.4) | 0.093 |
| HBsAg, positive | 198 (84.6) | 573 (85.3) | 0.809 |
| HBeAg, positive | 72 (30.8) | 190 (28.3) | 0.468 |
| HCV, positive | 10 (4.3) | 27 (4.0) | 0.865 |
| HBV-DNA level, > 2000 IU/mL | 92 (39.3) | 249 (37.1) | 0.538 |
| Preoperative antiviral therapy, yes | 15 (6.4) | 47 (7.0) | 0.761 |
| TBIL, µmol/L | 13.5 (4.4-52.8) | 13.6 (3.6-82.8) | 0.519 |
| ALB, g/L | 41.1 (34.0-53.8) | 40.1 (33.2-55.8) | 0.007 |
| ALT, IU/L | 35.9 (9.8-85.4) | 36.7 (7.7-86.1) | 0.420 |
| PT, seconds | 12.0 (10.8-15.7) | 12.2 (10.0-15.2) | 0.023 |
| PLT, 10^9^/L | 142.0 (81-419.0) | 148.0 (70.0-446.0) | 0.323 |
| AFP, ng/mL | 77.7 (0.8-55558.0) | 91.9(0.6-71210.0) | 0.144 |
| Hilar clamping, > 20 minutes | 182 (77.8) | 474 (70.5) | 0.033 |
| Blood transfusion, yes | 21 (9.0) | 64 (9.5) | 0.804 |
| Major hepatectomy*, yes | 84 (35.9) | 186 (27.7) | 0.018 |
| Cirrhosis^§^, yes | 101(43.2) | 314 (46.7) | 0.346 |
| Surgical margin^§^, > 1.0 cm | 131 (56.0) | 319 (47.5) | 0.025 |
| Tumour diameter^§^, cm | 4.2 (1.1-14.0) | 4.8 (1.1-15.0) | 0.268 |
| Tumour number^§^, multiple^†^ | 51 (21.8) | 168 (25.0) | 0.324 |
| Tumour capsule^§^, incomplete | 141 (60.3) | 385 (57.3) | 0.429 |
| MVI^§^, presence | 74 (31.6) | 244 (36.3) | 0.196 |
| Edmondson-Steiner grade^§^, III/IV | 169 (72.2) | 514 (76.5) | 0.192 |
| Surgical complication^‡^, grade III/IV | 19 (8.1) | 47 (7.0) | 0.568 |
| Adjuvant TACE, yes | 79 (33.8) | 237 (35.3) | 0.667 |
| **Abbreviations:** AR, anatomical resection; NAR, non-anatomical resection; BMI, body mass index; HBsAg, hepatitis B surface antigen; HBeAg, hepatitis B e antigen; HCV, hepatitis C virus; HBV-DNA, hepatitis B virus deoxyribonucleic acid; TBIL, total bilirubin; ALB, albumin; ALT, alanine transaminase; PT, prothrombin time; PLT, platelet; AFP, alpha fetoprotein; MVI, microvascular invasion; TACE, transarterial chemoembolization.  _*_: resection of three or more Couinaud’s hepatic segments.  §: based on postoperative pathology.  †: tumor nodules ≥ 2.  ‡: graded according to the Clavien-Dindo classification. | | | |
