# Supplementary material for: The Comparison of Surgical Margins and Type of Hepatic Resection for Hepatocellular Carcinoma With Microvascular Invasion
Source: Oncologist. 2023 May 17;28(11):e1043–51. doi: 10.1093/oncolo/oyad124 (PMC10628578; doi:10.1093/oncolo/oyad124)
Supplement: oyad124_suppl_Supplementary_Table_2 [file oyad124_suppl_supplementary_table_2.docx]

**Supplement Table 2. Univariable analysis of OS and TTR**

| **Variable** | **n** | **OS** | | |  | **TTR** | | |
| --- | --- | --- | --- | --- | --- | --- | --- | --- |
|  |  | ***P*** | **HR** | ***95%CI*** |  | ***P*** | **HR** | ***95%CI*** |
| Sex, male vs. female | 746/160 | .117 | 0.807 | 0.617-1.055 |  | .367 | 0.904 | 0.725-1.126 |
| ***Initial stage data*** |  |  |  |  |  |  |  |  |
| Age, years, > vs. ≤ 60 | 277/629 | .953 | 1.006 | 0.815-1.243 |  | .802 | 0.977 | 0.818-1.168 |
| BMI, ≥ vs. < 24 kg/m^2^ | 229/677 | .560 | 1.068 | 0.856-1.333 |  | .313 | 1.101 | 0.913-1.327 |
| Diabetes, yes vs. no | 60/846 | .241 | 1.249 | 0.861-1.812 |  | .565 | 1.101 | 0.793-1.531 |
| HBsAg, positive vs. negative | 771/135 | .292 | 1.164 | 0.878-1.543 |  | .375 | 1.111 | 0.880-1.402 |
| HBeAg, positive vs. negative | 262/644 | .970 | 1.004 | 0.810-1.244 |  | .278 | 1.104 | 0.923-1.322 |
| HCV, positive vs. negative | 37/869 | .502 | 1.171 | 0.739-1.855 |  | .260 | 1.249 | 0.848-1.838 |
| HBV-DNA, IU/mL, > vs. ≤ 2000 | 334/562 | .094 | 1.84 | 0.971-1.443 |  | .013 | 1.234 | 1.044-1.458 |
| Preoperative antiviral therapy, yes vs. no | 62/844 | .187 | 0.749 | 0.487-1.150 |  | .592 | 0.914 | 0.658-1.270 |
| TBIL, µmol/L, > vs. ≤ 17.1 | 205/701 | .820 | 1.027 | 0.815-1.294 |  | .566 | 1.059 | 0.871-1.287 |
| ALB, g/L, > vs. ≤ 35 | 852/54 | .100 | 0.679 | 0.428-1.076 |  | .785 | 0.954 | 0.677-1.342 |
| ALT, IU/L, > vs. ≤ 40 | 390/516 | .798 | 0.975 | 0.801-1.186 |  | .515 | 1.056 | 0.896-1.246 |
| PT, seconds, > vs. ≤ 12 | 494/412 | .144 | 1.157 | 0.951-1.408 |  | .059 | 1.173 | 0.994-1.383 |
| PLT, 10^9^/L, ≤ vs. > 100 | 179/727 | .018 | 0.723 | 0.553-0.945 |  | .016 | 0.763 | 0.612-0.951 |
| AFP, ng/mL, > vs. ≤ 200 | 566/340 | <.001 | 1.807 | 1.454-2.246 |  | <.001 | 1.511 | 1.267-1.802 |
| Hilar clamping, minutes, > vs. ≤ 20 | 656/250 | .719 | 1.041 | 0.836-1.297 |  | .202 | 1.130 | 0.937-1.363 |
| Blood transfusion, yes vs. no | 85/821 | .009 | 1.490 | 1.103-2.014 |  | .098 | 1.257 | 0.958-1.650 |
| Hepatectomy, AR vs. NAR | 234/672 | .063 | 1.243 | 0.988-1.564 |  | .068 | 1.194 | 0.987-1.443 |
| Hepatectomy, major* vs. minor | 270/636 | .946 | 1.007 | 0.816- 1.243 |  | .923 | 0.991 | 0.830-1.184 |
| Cirrhosis^§^, yes vs. no | 415/491 | .490 | 0.933 | 0.767-1.135 |  | .683 | 1.035 | 0.878-1.220 |
| Tumor diameter^§^, cm, > vs. ≤ 5 | 426/480 | <.001 | 2.404 | 1.962-2.946 |  | <.001 | 1.884 | 1.596-2.224 |
| Tumor number, multiple^†^ vs. single | 219/687 | <.001 | 2.396 | 1.960-2.929 |  | <.001 | 2.155 | 1.806-2.572 |
| Surgical margin^§^, cm, ≤ vs. >1.0 | 456/450 | <.001 | 1.489 | 1.224-1.812 |  | .001 | 1.338 | 1.135-1.577 |
| Tumour capsule^§^, incomplete vs. complete | 526/380 | <.001 | 1.746 | 1.420-2.147 |  | <.001 | 1.550 | 1.306-1.839 |
| MVI^§^, presence vs. absence | 318/588 | <.001 | 2.283 | 1.879-2.774 |  | <.001 | 1.852 | 1.569-2.186 |
| Edmondson-Steiner grade^§^, III/IV vs. I/II | 683/223 | <.001 | 1.767 | 1.368-2.282 |  | <.001 | 1.479 | 1.208-1.811 |
| Surgical complication grade^‡^, III/IV vs. I/II | 56/850 | .106 | 1.331 | 0.941-1.882 |  | .243 | 1.201 | 0.883-1.634 |
| Adjuvant TACE, yes vs. no | 316/590 | .515 | 1.069 | 0.875-1.307 |  | .893 | 1.012 | 0.853-1.200 |
| **Abbreviations:** OS, overall survival; HR, hazard ratio; CI, Confiden Intenral; TTR, time to recurrence; BMI, body mass index; HBsAg, hepatitis B surface antigen; HBeAg, hepatitis B e antigen; HCV, hepatitis C virus; HBV-DNA, hepatitis B virus deoxyribonucleic acid; TBIL, total bilirubin; ALB, albumin; ALT, alanine transaminase; PT, prothrombin time; PLT, platelet; AFP, alpha fetoprotein; AR, anatomical resection; NAR, non-anatomical resection; MVI, microvascular invasion; TACE, transarterial chemoembolization.  _*_: resection of 3 or more Couinaud’s hepatic segments.  §: based on postoperative pathology.  †: tumor nodules ≥ 2.  ‡: graded according to the Clavien-Dindo classification. | | | | | | | | |
