# Supplementary material for: The Comparison of Surgical Margins and Type of Hepatic Resection for Hepatocellular Carcinoma With Microvascular Invasion
Source: Oncologist. 2023 May 17;28(11):e1043–51. doi: 10.1093/oncolo/oyad124 (PMC10628578; doi:10.1093/oncolo/oyad124)
Supplement: oyad124_suppl_Supplementary_Table_3 [file oyad124_suppl_supplementary_table_3.docx]

**Supplement Table 3. Baseline characteristics of MVI-negative HCC patients**

| **Variable** | **Number (%)/median (range)** | | ***P*** |
| --- | --- | --- | --- |
|  | **AR group**  **(n=160)** | **NAR group**  **(n=428)** |  |
| Sex, male | 134 (83.8) | 345 (80.6) | 0.383 |
| ***Initial hepatectomy stage data*** |  |  |  |
| Age, years | 53.0 (24.0-79.0) | 53.0 (20.0-79.0) | 0.992 |
| BMI, ≥ 24 kg/m^2^ | 45 (28.1) | 116 (27.1) | 0.805 |
| Diabetes, yes | 7 (4.4) | 33 (7.7) | 0.153 |
| HBsAg, positive | 133 (83.1) | 361 (84.3) | 0.719 |
| HBeAg, positive | 49 (30.6) | 129 (30.1) | 0.909 |
| HCV, positive | 7 (4.4) | 19 (4.4) | 0.973 |
| HBV-DNA level, > 2000 IU/mL | 57 (35.6) | 155 (36.2) | 0.895 |
| Preoperative antiviral therapy, yes | 11 (6.9) | 31 (7.2) | 0.877 |
| TBIL, µmol/L | 13.2 (4.4-38.4) | 13.2 (3.6-82.8) | 0.702 |
| ALB, g/L | 41.4 (34.2-50.8) | 39.9 (33.2-54.8) | 0.002 |
| ALT, IU/L | 34.3 (9.8-83.2) | 37.3 (7.7-86.1) | 0.080 |
| PT, seconds | 12.0 (10.8-15.7) | 12.2 (10.5-15.0) | 0.010 |
| PLT, 10^9^/L | 147.0 (81-367.0) | 151.0 (70.0-446.0) | 0.287 |
| AFP, ng/mL | 49.3 (0.8-55558.0) | 59.9(0.6-71210.0) | 0.367 |
| Hilar clamping, > 20 minutes | 123 (76.9) | 298 (69.6) | 0.083 |
| Blood transfusion, yes | 13 (8.1) | 37 (8.6) | 0.841 |
| Major hepatectomy*, yes | 65 (40.6) | 118 (27.6) | 0.002 |
| Cirrhosis^§^, yes | 70(43.8) | 189 (44.2) | 0.929 |
| Surgical margin^§^, > 1.0 cm | 100 (62.5) | 210 (49.1) | 0.004 |
| Tumour diameter^§^, cm | 4.0 (1.1-14.0) | 4.4 (1.1-15.0) | 0.623 |
| Tumour number^§^, multiple^†^ | 32 (20.0) | 86 (20.1) | 0.980 |
| Tumour capsule^§^, incomplete | 91 (56.9) | 200 (46.7) | 0.029 |
| Edmondson-Steiner grade^§^, III/IV | 107 (66.0) | 309 (72.2) | 0.207 |
| Surgical complication grade ^‡^, III/IV | 14 (8.8) | 30 (7.0) | 0.475 |
| Adjuvant TACE, yes | 45 (28.1) | 125 (29.2) | 0.797 |
| **Abbreviations:** AR, anatomical resection; NAR, non-anatomical resection; BMI, body mass index; HBsAg, hepatitis B surface antigen; HBeAg, hepatitis B e antigen; HCV, hepatitis C virus; HBV-DNA, hepatitis B virus deoxyribonucleic acid; TBIL, total bilirubin; ALB, albumin; ALT, alanine transaminase; PT, prothrombin time; PLT, platelet; AFP, alpha fetoprotein; MVI, microvascular invasion; TACE, transarterial chemoembolization.  _*_: resection of 3 or more Couinaud’s hepatic segments.  §: based on postoperative pathology  †: tumour nodules ≥ 2.  ‡: graded according to the Clavien-Dindo classification. | | | |
