# Supplementary material for: The Comparison of Surgical Margins and Type of Hepatic Resection for Hepatocellular Carcinoma With Microvascular Invasion
Source: Oncologist. 2023 May 17;28(11):e1043–51. doi: 10.1093/oncolo/oyad124 (PMC10628578; doi:10.1093/oncolo/oyad124)
Supplement: oyad124_suppl_Supplementary_Table_4 [file oyad124_suppl_supplementary_table_4.docx]

**Supplement Table 4. Univariable analysis of OS and TTR of MVI-negative HCC patients**

| **Variable** | **n** | **OS** | | |  | **TTR** | | |
| --- | --- | --- | --- | --- | --- | --- | --- | --- |
|  |  | ***P*** | **HR** | ***95%CI*** |  | ***P*** | **HR** | ***95%CI*** |
| Sex, male vs. female | 349/73 | .738 | 0.941 | 0.659-1.343 |  | .927 | 0.987 | 0.744-1.309 |
| ***Initial stage data*** |  |  |  |  |  |  |  |  |
| Age, years, > vs. ≤ 60 | 130/292 | .122 | 1.252 | 0.942-1.663 |  | .599 | 1.064 | 0.844-1.342 |
| BMI, ≥ vs. < 24 kg/m^2^ | 109/313 | .080 | 1.300 | 0.969-1.744 |  | .058 | 1.259 | 0.992-1.599 |
| Diabetes, yes vs. no | 23/399 | .452 | 1.223 | 0.723-2.069 |  | .624 | 1.114 | 0.723-1.718 |
| HBsAg, positive vs. negative | 345/77 | .273 | 1.246 | 0.841-1.844 |  | .133 | 1.271 | 0.930-1.736 |
| HBeAg, positive vs. negative | 134/288 | .984 | 0.997 | 0.741-1.340 |  | .235 | 1.151 | 0.912-1.453 |
| HCV, positive vs. negative | 20/402 | .762 | 1.103 | 0.584-2.083 |  | .306 | 1.291 | 0.792-2.104 |
| HBV-DNA, IU/mL, > vs. ≤ 2000 | 161/261 | .793 | 1.039 | 0.783-1.378 |  | .182 | 1.164 | 0.931-1.456 |
| Preoperative antiviral therapy, yes vs. no | 27/395 | .325 | 0.737 | 0.402-1.353 |  | .952 | 1.013 | 0.663-1.548 |
| TBIL, µmol/L, > vs. ≤ 17.1 | 91/331 | .536 | 0.898 | 0.638-1.264 |  | .928 | 1.012 | 0.775-1.323 |
| ALB, g/L, > vs. ≤ 35 | 401/21 | .248 | 0.687 | 0.364-1.298 |  | .888 | 1.032 | 0.669-1.590 |
| ALT, IU/L, > vs. ≤ 40 | 177/245 | .855 | 0.975 | 0.740-1.283 |  | .301 | 1.123 | 0.902-1.397 |
| PT, seconds, > vs. ≤ 12 | 219/203 | .405 | 1.124 | 0.854-1.480 |  | .197 | 1.155 | 0.928-1.439 |
| PLT, 10^9^/L, ≤ vs. > 100 | 86/336 | .102 | 0.727 | 0.496-1.065 |  | .205 | 0.829 | 0.620-1.108 |
| AFP, ng/mL, > vs. ≤ 200 | 247/175 | .001 | 1.648 | 1.230-2.208 |  | .001 | 1.459 | 1.163-1.832 |
| Hepatectomy, AR vs. NAR | 211/211 | .596 | 1.088 | 0.797-1.483 |  | .561 | 1.074 | 0.841-1.372 |
| Hepatectomy, major* vs. minor | 134/288 | .812 | 1.036 | 0.775- 1.385 |  | .669 | 0.950 | 0.751-1.202 |
| Hilar clamping, minutes, > 20 vs. ≤20 | 316/106 | .876 | 1.025 | 0.754-1.393 |  | .502 | 1.089 | 0.850-1.395 |
| Tumour diameter^§^, cm, > vs. ≤ 5 | 193/229 | <.001 | 2.303 | 1.738-3.050 |  | <.001 | 1.739 | 1.398-2.164 |
| Tumour number^§^, multiple^†^ vs. single | 102/320 | <.001 | 2.301 | 1.722-3.075 |  | <.001 | 2.197 | 1.724-2.801 |
| Surgical margin^§^, cm, ≤ vs. >1.0 | 203/219 | .741 | 0.955 | 0.727-1.255 |  | .889 | 1.016 | 0.816-1.264 |
| Tumour capsule^§^, incomplete vs. complete | 258/164 | <.001 | 1.663 | 1.260-2.195 |  | <.001 | 1.508 | 1.210-1.879 |
| Edmondson-Steiner grade^§^, III/IV vs. I/II | 312/110 | .026 | 1.442 | 1.044-1.991 |  | .084 | 1.243 | 0.971-1.591 |
| Cirrhosis^§^, yes vs. no | 192 /230 | .715 | 0.950 | 0.720-1.252 |  | .526 | 1.074 | 0.862-1.337 |
| Blood transfusion, yes vs. no | 41/381 | .021 | 1.652 | 1.079-2.528 |  | .194 | 1.283 | 0.881-1.868 |
| Surgical complication grade^‡^, III/IV vs. I/II | 24/398 | .122 | 1.439 | 0.907-2.282 |  | .138 | 1.341 | 0.910-1.975 |
| Adjuvant treatment, yes vs. no | 137/285 | .493 | 0.899 | 0.664-1.218 |  | .220 | 0.859 | 0.673-1.095 |
| **Abbreviations:** OS, overall survival; HR, hazard ratio; CI, Confiden Intenral; TTR, time to recurrence; BMI, body mass index; HBsAg, hepatitis B surface antigen; HBeAg, hepatitis B e antigen; HCV, hepatitis C virus; HBV-DNA, hepatitis B virus deoxyribonucleic acid; TBIL, total bilirubin; ALB, albumin; ALT, alanine transaminase; PT, prothrombin time; PLT, platelet; AFP, alpha fetoprotein; MVI, microvascular invasion; TACE, transarterial chemoembolization.  _*_: resection of three or more Couinaud’s hepatic segments.  §: based on postoperative pathology.  †: tumour nodules ≥ 2.  ‡: graded according to the Clavien-Dindo classification. | | | | | | | | |
