# Supplementary material for: The Comparison of Surgical Margins and Type of Hepatic Resection for Hepatocellular Carcinoma With Microvascular Invasion
Source: Oncologist. 2023 May 17;28(11):e1043–51. doi: 10.1093/oncolo/oyad124 (PMC10628578; doi:10.1093/oncolo/oyad124)
Supplement: oyad124_suppl_Supplementary_Table_5 [file oyad124_suppl_supplementary_table_5.docx]

**Supplement Table 5. Baseline characteristics of MVI-positive HCC patients**

| **Variable** | **Number (%)/median (range)** | | ***P*** |
| --- | --- | --- | --- |
|  | **AR group**  **(n=74)** | **NAR group**  **(n=244)** |  |
| Sex, male | 61 (82.4) | 206 (84.4) | 0.682 |
| ***Initial hepatectomy stage data*** |  |  |  |
| Age, years | 54.0 (18.0-75.0) | 51.0 (18.0-80.0) | 0.139 |
| BMI, ≥ 24 kg/m^2^ | 21 (28.4) | 47 (19.3) | 0.094 |
| Diabetes, yes | 3 (4.1) | 17 (7.0) | 0.528 |
| HBsAg, positive | 65 (87.8) | 212 (86.9) | 0.830 |
| HBeAg, positive | 23 (31.1) | 61 (25.0) | 0.299 |
| HCV, positive | 3 (4.1) | 8 (3.3) | 1.000 |
| HBV-DNA level, > 2000 IU/mL | 35 (47.3) | 94 (38.5) | 0.178 |
| Preoperative antiviral therapy, yes | 4 (5.4) | 16 (6.6) | 0.933 |
| TBIL, µmol/L | 14.1 (4.5-52.8) | 14.0 (5.1-38.3) | 0.606 |
| ALB, g/L | 40.3 (34.0-53.8) | 40.7 (33.3-55.8) | 0.782 |
| ALT, IU/L | 39.3 (13.9-85.4) | 35.4 (9.8-80.4) | 0.273 |
| PT, seconds | 12.1 (10.9-15.3) | 12.1 (10.0-15.2) | 0.956 |
| PLT, 10^9^/L | 136.0 (81-419.0) | 143.0 (70.0-426.0) | 0.797 |
| AFP, ng/mL | 128.5 (0.9-32210.0) | 221.1(0.6-61210.0) | 0.362 |
| Hilar clamping, > 20 minutes | 59 (79.7) | 176 (72.1) | 0.192 |
| Blood transfusion, yes | 8 (10.8) | 27 (11.1) | 0.951 |
| Major hepatectomy*, yes | 19 (25.7) | 68 (27.9) | 0.711 |
| Cirrhosis^§^, yes | 31(41.9) | 125 (51.2) | 0.159 |
| Surgical margin^§^, > 1.0 cm | 31 (41.9) | 109 (44.7) | 0.673 |
| Tumour diameter^§^, cm | 4.7 (1.1-14.0) | 5.2 (1.1-14.5) | 0.373 |
| Tumour number^§^, multiple^†^ | 19 (25.7) | 82 (33.6) | 0.199 |
| Tumour capsule^§^, incomplete | 50 (67.6) | 185 (75.8) | 0.157 |
| Edmondson-Steiner grade^§^, III/IV | 62 (83.8) | 205 (84.0) | 0.962 |
| Surgical complication grade ^‡^, III/IV | 5 (6.8) | 17 (7.0) | 0.950 |
| Adjuvant TACE, yes | 34 (45.9) | 112 (45.9) | 0.995 |
| **Abbreviations:** AR, anatomical resection; NAR, non-anatomical resection; BMI, body mass index; HBsAg, hepatitis B surface antigen; HBeAg, hepatitis B e antigen; HCV, hepatitis C virus; HBV-DNA, hepatitis B virus deoxyribonucleic acid; TBIL, total bilirubin; ALB, albumin; ALT, alanine transaminase; PT, prothrombin time; PLT, platelet; AFP, alpha fetoprotein; MVI, microvascular invasion; TACE, transarterial chemoembolization.  _*_: resection of 3 or more Couinaud’s hepatic segments.  §: based on postoperative pathology  †: tumour nodules ≥ 2.  ‡: graded according to the Clavien-Dindo classification. | | | |
