# Supplementary material for: The Comparison of Surgical Margins and Type of Hepatic Resection for Hepatocellular Carcinoma With Microvascular Invasion
Source: Oncologist. 2023 May 17;28(11):e1043–51. doi: 10.1093/oncolo/oyad124 (PMC10628578; doi:10.1093/oncolo/oyad124)
Supplement: oyad124_suppl_Supplementary_Table_6 [file oyad124_suppl_supplementary_table_6.docx]

**Supplement Table 6. Univariable analysis of OS and TTR of MVI-positive HCC patients**

| **Variable** | **n** | **OS** | | |  | **TTR** | | |
| --- | --- | --- | --- | --- | --- | --- | --- | --- |
|  |  | ***P*** | **HR** | ***95%CI*** |  | ***P*** | **HR** | ***95%CI*** |
| Sex, male vs. female | 349/73 | .076 | 0.689 | 0.456-1.040 |  | .282 | 0.824 | 0.578-1.173 |
| ***Initial stage data*** |  |  |  |  |  |  |  |  |
| Age, years, > vs. ≤ 60 | 130/292 | .209 | 0.815 | 0.592-1.122 |  | .492 | 0.906 | 0.684-1.201 |
| BMI, ≥ vs. < 24 kg/m^2^ | 109/313 | .694 | 0.933 | 0.662-1.316 |  | .783 | 0.958 | 0.707-1.299 |
| Diabetes, yes vs. no | 23/399 | .326 | 1.302 | 0.769-2.203 |  | .697 | 1.106 | 0.666-1.836 |
| HBsAg, positive vs. negative | 345/77 | .779 | 0.944 | 0.629-1.416 |  | .182 | 0.788 | 0.555-1.118 |
| HBeAg, positive vs. negative | 134/288 | .380 | 1.150 | 0.842-1.571 |  | .315 | 1.157 | 0.871-1.538 |
| HCV, positive vs. negative | 20/402 | .267 | 1.460 | 0.748-2.849 |  | .443 | 1.281 | 0.680-2.412 |
| HBV-DNA, IU/mL, > vs. ≤ 2000 | 161/261 | .060 | 1.306 | 0.989-1.726 |  | .030 | 1.321 | 1.027-1.699 |
| Preoperative antiviral therapy, yes vs. no | 27/395 | .391 | 0.766 | 0.417-1.408 |  | .309 | 0.762 | 0.452-1.285 |
| TBIL, µmol/L, > vs. ≤ 17.1 | 91/331 | .540 | 1.104 | 0.805-1.514 |  | .661 | 1.066 | 0.801-1.420 |
| ALB, g/L, > vs. ≤ 35 | 401/21 | .278 | 0.691 | 0.354-1.348 |  | .650 | 0.879 | 0.503-1.536 |
| ALT, IU/L, > vs. ≤ 40 | 177/245 | .880 | 0.979 | 0.740-1.294 |  | .823 | 0.972 | 0.756-1.250 |
| PT, seconds, > vs. ≤ 12 | 219/203 | .402 | 1.126 | 0.853-1.488 |  | .353 | 1.126 | 0.877-1.446 |
| PLT, 10^9^/L, ≤ vs. > 100 | 86/336 | .049 | 0.684 | 0.469-0.998 |  | .012 | 0.649 | 0.463-0.911 |
| AFP, ng/mL, > vs. ≤ 200 | 247/175 | .001 | 1.718 | 1.236-2.388 |  | .032 | 1.357 | 1.024-1.799 |
| Hepatectomy, AR vs. NAR | 211/211 | .048 | 1.414 | 1.003-1.993 |  | .043 | 1.367 | 1.010-1.849 |
| Hepatectomy, major* vs. minor | 134/288 | .650 | 1.074 | 0.790- 1.459 |  | .295 | 1.157 | 0.881-1.521 |
| Hilar clamping, minutes, > 20 vs. ≤20 | 316/106 | .816 | 1.038 | 0.757-1.424 |  | .276 | 1.173 | 0.880-1.564 |
| Tumour diameter^§^, cm, > vs. ≤ 5 | 193/229 | <.001 | 2.342 | 1.743-3.148 |  | <.001 | 2.002 | 1.545-2.594 |
| Tumour number^§^, multiple^†^ vs. single | 102/320 | <.001 | 2.131 | 1.609-2.822 |  | <.001 | 1.809 | 1.396-2.344 |
| Surgical margin^§^, cm, ≤ vs. >1.0 | 203/219 | <.001 | 2.525 | 1.875-3.401 |  | <.001 | 1.962 | 1.514-2.544 |
| Tumour capsule^§^, incomplete vs. complete | 258/164 | .196 | 1.237 | 0.896-1.707 |  | .365 | 1.140 | 0.858-1.515 |
| Edmondson-Steiner grade^§^, III/IV vs. I/II | 312/110 | .004 | 1.886 | 1.221-2.913 |  | .006 | 1.683 | 1.161-2.442 |
| Cirrhosis^§^, yes vs. no | 192 /230 | .181 | 0.827 | 0.626-1.092 |  | .357 | 0.889 | 0.693-1.141 |
| Blood transfusion, yes vs. no | 41/381 | .390 | 1.206 | 0.787-1.847 |  | .549 | 1.128 | 0.761-1.671 |
| Surgical complication grade^‡^, III/IV vs. I/II | 24/398 | .295 | 1.325 | 0.783-2.244 |  | .955 | 1.015 | 0.611-1.685 |
| Adjuvant treatment, yes vs. no | 137/285 | .547 | 0.918 | 0.696-1.212 |  | .828 | 0.973 | 0.758-1.248 |
| **Abbreviations:** OS, overall survival; HR, hazard ratio; CI, Confiden Intenral; TTR, time to recurrence; BMI, body mass index; HBsAg, hepatitis B surface antigen; HBeAg, hepatitis B e antigen; HCV, hepatitis C virus; HBV-DNA, hepatitis B virus deoxyribonucleic acid; TBIL, total bilirubin; ALB, albumin; ALT, alanine transaminase; PT, prothrombin time; PLT, platelet; AFP, alpha fetoprotein; AR, anatomical resection; NAR, non-anatomical resection; MVI, microvascular invasion; TACE, transarterial chemoembolization.  _*_: resection of 3 or more Couinaud’s hepatic segments.  §: based on postoperative pathology.  †: tumour nodules ≥ 2.  ‡: graded according to the Clavien-Dindo classification. | | | | | | | | |
