# Supplementary material for: The Comparison of Surgical Margins and Type of Hepatic Resection for Hepatocellular Carcinoma With Microvascular Invasion
Source: Oncologist. 2023 May 17;28(11):e1043–51. doi: 10.1093/oncolo/oyad124 (PMC10628578; doi:10.1093/oncolo/oyad124)
Supplement: oyad124_suppl_Supplementary_Table_7 [file oyad124_suppl_supplementary_table_7.docx]

**Supplement Table 7. Baseline characteristics of MVI-positive HCC patients underwent AR with narrow margin or NAR with wide margin**

| **Variable** | **Number (%)/median (range)** | | ***P*** |
| --- | --- | --- | --- |
|  | **AR with narrow margin (n=43)** | **NAR with wide margin**  **(n=109)** |  |
| Sex, male | 38 (88.4) | 88 (80.7) | 0.260 |
| ***Initial hepatectomy stage data*** |  |  |  |
| Age, years | 54.0 (28.0-69.0) | 48.0 (20.0-75.0) | 0.127 |
| BMI, ≥ 24 kg/m^2^ | 10 (23.3) | 16 14.7) | 0.206 |
| Diabetes, yes | 1 (2.3) | 5 (4.6) | 0.855 |
| HBsAg, positive | 36 (83.7) | 92 (84.4) | 0.917 |
| HBeAg, positive | 12 (27.9) | 28 (25.7) | 0.780 |
| HCV, positive | 3(7.0) | 3 (2.8) | 0.352 |
| HBV-DNA level, > 2000 IU/mL | 19 (44.2) | 48 (44.0) | 0.987 |
| Preoperative antiviral therapy, yes | 2 (4.7) | 3 (2.8) | 0.931 |
| TBIL, µmol/L | 14.2 (4.5-52.8) | 12.9 (5.1-32.0) | 0.270 |
| ALB, g/L | 40.4 (34.0-52.3) | 41.0 (33.3-54.1) | 1.000 |
| ALT, IU/L | 41.0 (14.3-85.4) | 35.0 (11.1-79.1) | 0.173 |
| PT, seconds | 12.1 (10.9-14.9) | 12.0 (10.0-15.0) | 0.630 |
| PLT, 10^9^/L | 138.0 (81-402.0) | 146.0 (72.0-426.0) | 0.820 |
| AFP, ng/mL | 319.1(1.7-32210.0) | 114.0(0.6-44210.0) | 0.375 |
| Hilar clamping, > 20 minutes | 35 (81.4) | 73 (67.0) | 0.077 |
| Blood transfusion, yes | 6 (14.0) | 10 (9.2) | 0.387 |
| Major hepatectomy*, yes | 12 (27.9) | 34 (31.2) | 0.691 |
| Cirrhosis^§^, yes | 18(41.9) | 56 (51.4) | 0.290 |
| Tumour diameter^§^, cm | 6.0 (1.2-14.0) | 5.2 (1.4-14.2) | 0.394 |
| Tumour number^§^, multiple^†^ | 14 (32.6) | 28 (25.7) | 0.394 |
| Tumour capsule^§^, incomplete | 29 (67.4) | 81 (74.3) | 0.394 |
| Edmondson-Steiner grade^§^, III/IV | 39 (90.7) | 86 (78.9) | 0.086 |
| Surgical complication grade ^‡^, III/IV | 3 (7.0) | 9 (8.3) | 1.000 |
| Adjuvant TACE, yes | 19 (44.2) | 40 (36.7) | 0.393 |
| **Abbreviations:** AR, anatomical resection; NAR, non-anatomical resection; BMI, body mass index; HBsAg, hepatitis B surface antigen; HBeAg, hepatitis B e antigen; HCV, hepatitis C virus; HBV-DNA, hepatitis B virus deoxyribonucleic acid; TBIL, total bilirubin; ALB, albumin; ALT, alanine transaminase; PT, prothrombin time; PLT, platelet; AFP, alpha fetoprotein; MVI, microvascular invasion; TACE, transarterial chemoembolization.  _*_: resection of 3 or more Couinaud’s hepatic segments.  §: based on postoperative pathology  †: tumour nodules ≥ 2.  ‡: graded according to the Clavien-Dindo classification. | | | |
