# Supplementary material for: The Comparison of Surgical Margins and Type of Hepatic Resection for Hepatocellular Carcinoma With Microvascular Invasion
Source: Oncologist. 2023 May 17;28(11):e1043–51. doi: 10.1093/oncolo/oyad124 (PMC10628578; doi:10.1093/oncolo/oyad124)
Supplement: oyad124_suppl_Supplementary_Table_8 [file oyad124_suppl_supplementary_table_8.docx]

**Supplement Table 8. Univariable analysis of OS and TTR for MVI-positive HCC patients underwent AR with narrow margin or NAR with wide margin**

| **Variable** | **n** | **OS** | | |  | **TTR** | | |
| --- | --- | --- | --- | --- | --- | --- | --- | --- |
|  |  | ***P*** | **HR** | ***95%CI*** |  | ***P*** | **HR** | ***95%CI*** |
| Sex, male vs. female | 749/157 | .189 | 0.664 | 0.361-1.224 |  | .659 | 0.893 | 0.540-1.477 |
| ***Initial stage data*** |  |  |  |  |  |  |  |  |
| Age, years, > vs. ≤ 60 | 270/636 | .470 | 1.190 | 0.743-1.907 |  | .516 | 1.148 | 0.757-1.741 |
| BMI, ≥ vs. < 24 Kg/m^2^ | 225/681 | .290 | 0.719 | 0.391-1.325 |  | .864 | 0.958 | 0.585-1.567 |
| Diabetes, yes vs. no | 58/848 | .262 | 1.677 | 0.679-4.141 |  | .069 | 2.155 | 0.943-4.923 |
| HBsAg, positive vs. negative | 775/131 | .915 | 1.032 | 0.572-1.863 |  | .166 | 0.718 | 0.449-1.148 |
| HBeAg, positive vs. negative | 260/646 | .952 | 1.015 | 0.629-1.636 |  | .436 | 1.178 | 0.780-1.780 |
| HBV-DNA, IU/mL, > vs. ≤ 2000 | 337/569 | .171 | 1.346 | 0.879-2.062 |  | .704 | 1.075 | 0.740-1.562 |
| Antiviral therapy, yes vs. no | 62/844 | .188 | 0.266 | 0.037-1.909 |  | .264 | 0.520 | 0.165-1.638 |
| TBIL, µmol/L, > vs. ≤ 17.1 | 205/701 | .124 | 0.620 | 0.337-1.141 |  | .211 | 0.730 | 0.446-1.196 |
| ALB, g/L, > vs. ≤ 35 | 852/54 | .565 | 0.767 | 0.311-1.893 |  | .762 | 0.889 | 0.414-1.909 |
| ALT, IU/L, > vs. ≤ 40 | 390/516 | .425 | 0.839 | 0.546-1.291 |  | .618 | 0.910 | 0.629-1.317 |
| PT, seconds, > vs. ≤ 12 | 499/407 | .876 | 1.034 | 0.678-1.579 |  | .415 | 1.165 | 0.807-1.680 |
| PLT, 10^9^/L, ≤ vs. > 100 | 179/727 | .331 | 0.753 | 0.425-1.335 |  | .170 | 0.708 | 0.433-1.159 |
| AFP, ng/mL, > vs. ≤ 200 | 588/318 | .033 | 1.724 | 1.044-2.845 |  | .500 | 1.149 | 0.767-1.723 |
| Hepatectomy, AR with narrow margin vs. NAR with wide margin | 234/672 | .008 | 0.551 | 0.355-0.857 |  | .024 | 0.638 | 0.432-0.942 |
| Hepatectomy, major* vs. minor | 265/641 | .889 | 0.967 | 0.607- 1.541 |  | .502 | 1.145 | 0.772-1.698 |
| Hilar clamping, minutes, > 20 vs. ≤20 | 670/236 | .664 | 1.112 | 0.690-1.793 |  | .260 | 1.271 | 0.837-1.929 |
| Tumour diameter^§^, cm, > vs. ≤ 5 | 428/478 | .003 | 1.961 | 1.253-3.069 |  | .016 | 1.585 | 1.089-2.308 |
| Tumour number^§^, multiple^†^ vs. single | 219/687 | .001 | 2.053 | 1.328-3.173 |  | .081 | 1.427 | 0.958-2.126 |
| Surgical margin^§^, cm, ≤ vs. >1.0 | 476/430 | .008 | 1.814 | 1.167-2.821 |  | .024 | 1.568 | 1.062-2.315 |
| Tumour capsule^§^, incomplete vs. complete | 520/386 | .295 | 1.296 | 0.798-2.104 |  | .635 | 1.103 | 0.736-1.653 |
| Edmondson-Steiner grade^§^, III/IV vs. I/II | 683/223 | .303 | 1.364 | 0.756-2.461 |  | .473 | 1.198 | 0.732-1.960 |
| Cirrhosis^§^, yes vs. no | 415/491 | .149 | 0.730 | 0.476-1.120 |  | .237 | 0.801 | 0.554-1.157 |
| Blood transfusion, yes vs. no | 84/822 | .925 | 1.967 | 0.485-1.930 |  | .696 | 0.883 | 0.474-1.645 |
| Surgical complication grade^‡^, III/IV vs. I/II | 56/850 | .541 | 0.698 | 0.220-2.209 |  | .551 | 0.738 | 0.272-2.003 |
| Adjuvant treatment, yes vs. no | 316/590 | .985 | 0.996 | 0.649-1.529 |  | .585 | 1.109 | 0.765-1.606 |
| **Abbreviations:** OS, overall survival; HR, hazard ratio; CI, Confiden Intenral; TTR, time to recurrence; BMI, body mass index; HBsAg, hepatitis B surface antigen; HBeAg, hepatitis B e antigen; HCV, hepatitis C virus; HBV-DNA, hepatitis B virus deoxyribonucleic acid; TBIL, total bilirubin; ALB, albumin; ALT, alanine transaminase; PT, prothrombin time; PLT, platelet; AFP, alpha fetoprotein; AR, anatomical resection; NAR, non-anatomical resection; MVI, microvascular invasion; TACE, transarterial chemoembolization.  _*_: resection of 3 or more Couinaud’s hepatic segments.  §: based on postoperative pathology.  †: tumour nodules ≥ 2.  ‡: graded according to the Clavien-Dindo classification. | | | | | | | | |
